# Design and assessment of TRAP-CSP fusion antigens as effective malaria vaccines

**DOI:** 10.1371/journal.pone.0216260

**Published:** 2020-01-22

**Authors:** Chafen Lu, Gaojie Song, Kristin Beale, Jiabin Yan, Emma Garst, Juan Feng, Emily Lund, Flaminia Catteruccia, Timothy A. Springer

**Affiliations:** 1 Program in Cellular and Molecular Medicine, Boston Children’s Hospital, Harvard Medical School, Boston, Massachusetts, United States of America; 2 Department of Immunology and Infectious Diseases, Harvard T.H. Chan School of Public Health, Boston, Massachusetts, United States of America; Ehime Daigaku, JAPAN

## Abstract

The circumsporozoite protein (CSP) and thrombospondin-related adhesion protein (TRAP) are major targets for pre-erythrocytic malaria vaccine development. However, the CSP-based vaccine RTS,S provides only marginal protection, highlighting the need for innovative vaccine design and development. Here we design and characterize expression and folding of *P*. *berghei* (*Pb*) and *P*. *falciparum* (*Pf)* TRAP-CSP fusion proteins, and evaluate immunogenicity and sterilizing immunity in mice. TRAP N-terminal domains were fused to the CSP C-terminal αTSR domain with or without the CSP repeat region, expressed in mammalian cells, and evaluated with or without N-glycan shaving. *Pb* and *Pf* fusions were each expressed substantially better than the TRAP or CSP components alone; furthermore, the fusions but not the CSP component could be purified to homogeneity and were well folded and monomeric. As yields of TRAP and CSP fragments were insufficient, we immunized BALB/c mice with *Pb* TRAP-CSP fusions in AddaVax adjuvant and tested the effects of absence or presence of the CSP repeats and absence or presence of high mannose N-glycans on total antibody titer and protection from infection by mosquito bite both 2.5 months and 6 months after the last immunization. Fusions containing the repeats were completely protective against challenge and re-challenge, while those lacking repeats were significantly less effective. These results correlated with higher total antibody titers when repeats were present. Our results show that TRAP-CSP fusions increase protein antigen production, have the potential to yield effective vaccines, and also guide design of effective proteins that can be encoded by nucleic acid-based and virally vectored vaccines.

## Introduction

Malaria remains a global health problem with an estimated 216 million cases of infection and 445,000 deaths worldwide in 2016. Children under age 5 are most vulnerable and suffer high mortality. Malaria is caused by *Plasmodium* parasites transmitted by *Anopheles* mosquitoes. Infected mosquitoes introduce salivary gland sporozoites into the host during a blood meal. Sporozoites infect hepatocytes, and subsequent infection of red blood cells causes the symptoms of malaria.

To date there are no effective malaria vaccines. Most vaccine development has targeted the pre-erythrocytic stage, i.e., liver infection. The most advanced pre-erythrocytic subunit vaccine, RTS,S, in a phase III trial reduced infection by only 27% in infants and 46% in children during the first 18 months. Infection rates increased thereafter [1, 2]. Although immunizations with live *P*. *falciparum* sporozoites, attenuated by radiation or mutation or given in combination with chemoprophylaxis, have provided 50 to >90% protection against challenge with a laboratory malaria strain in controlled human malaria infection [3–7], the protective efficacy of *P*. *falciparum* sporozoite (*Pf*SPZ) vaccine against malaria infection in the field in a phase I trial was less than in controlled human malaria infection studies [8].

**Fig 1:**
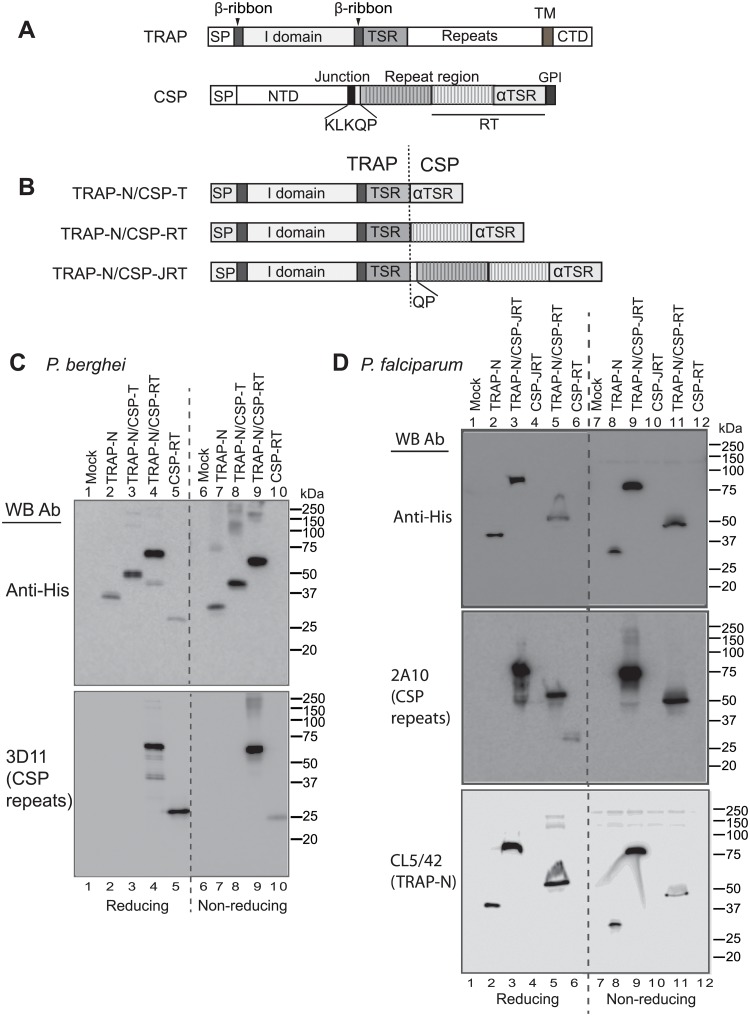
TRAP-CSP fusion antigen design and expression. (A and B) Schematic diagrams. SP, signal peptide; TM, transmembrane domain; CTD, cytoplasmic domain; NTD, N-terminal domain; GPI, glycosylphosphatidylinositol membrane anchor. The region included in the RTS component of RTS,S vaccine is shown under the CSP diagram. The vertical dashed line shows the fusion junction between TRAP and CSP. (C) and (D) Expression in 293T transfectants of *P*. *berghei* constructs (C) and *P*. *falciparum* constructs (D). Supernatants from 293T cells transiently transfected with the indicated constructs or vector alone (mock) were subjected to reducing or nonreducing SDS 10% PAGE and Western blot with antibodies as indicated.

Two major proteins on the surface of sporozoites, circumsporozoite protein (CSP) and thrombospondin-related adhesion protein (TRAP), are a focus of pre-erythrocytic subunit vaccine development; both are essential for sporozoite motility and liver-stage infection [9, 10]. CSP, the most abundant surface protein in sporozoites, is composed of an N-terminal domain (NTD), a so-called Region I (RI) or junction region where CSP is cleaved during cell invasion, a central repeat region, an αTSR domain, and a C-terminal glycosylphosphatidylinositol (GPI) membrane anchor [11–13] (Fig 1A). The RTS,S vaccine includes the C-terminal portion of the repeat region (R) and the αTSR domain (T) from *P*. *falciparum* CSP (RT, Fig 1A) fused to the hepatitis B surface antigen (S) as the RTS component and an excess of S [14].

TRAP contains an N-terminal domain that is commonly referred to as von Willebrand factor A domain, although it is most similar to an integrin I domain because it contains a metal ion-dependent adhesion site (MIDAS) with a bound Mg^2+^ ion that is required for sporozoite motility in vitro and infection in vivo [15, 16]. The I domain is inserted in an extensible β-ribbon and followed by a thrombospondin repeat (TSR) domain, C-terminal repeats, a single-pass transmembrane domain, and a cytoplasmic domain (Fig 1A).

Recent TRAP-based vaccine development has largely focused on viral-vectored ME-TRAP, which encodes multiple T cell epitopes (ME) from *Plasmodium* and TRAP [17] [18]. ME-TRAP delivered by adenovirus prime and modified vaccinia Ankara virus (MVA) boost achieved 21% sterile protection in controlled human malaria infection [19], and in a field trial, reduced infection risk in Kenyan adults by 67% based on PCR detection of blood *P*. *falciparum* [20]. However, no significant protective efficacy was observed in two recent field trials [21, 22].

Combination vaccination targeting both CSP and TRAP has also been explored. A phase I/II clinical trial combining adjuvanted TRAP protein with RTS,S showed no benefit compared to RTS,S alone [23], however, combining a modified RTS,S-like vaccine R21 with viral-vectored TRAP significantly enhanced preclinical protective efficacy compared to single component vaccines in BALB/c mice [24]. Combining CSP in protein or viral vectored forms with viral vectored TRAP has variously given interference or additivity in mice [25]. Additionally, co-administration of viral-vectored ME-TRAP with adjuvanted RTS,S reduced immunogenicity and efficacy to RTS,S alone in controlled human malaria infection [26].

Previous studies reviewed above used CSP in viral-vectored or protein form combined with a separate viral-vectored TRAP. Furthermore, CSP in RTS,S or R21 has not been demonstrated to have a folded αTSR domain. Additionally, TRAP and CSP encoded by viral vectors have not been examined for extent of expression in host cells or folding.

Here, we have attempted to improve combined TRAP-CSP vaccines in multiple ways. We fused an N-terminal fragment of TRAP with C-terminal fragments of CSP. We demonstrate that both *Pb* and *Pf* fusions are better expressed than the individual components alone, and with antibodies to *Pf* CSP and with antibodies that we report here to *Pf* TRAP, we demonstrate that their domains are well folded. With *Pb* fusions we demonstrated long-lived, protective immunity. The fusion proteins have the potential to enable synergy between epitopes on CSP and TRAP, although our yields of CSP and TRAP fragments alone were insufficient to experimentally test this idea. Good antigen expression is necessary not only for protein antigen vaccines, but also for modified RNA or viral vector vaccines, and thus our results are relevant to further development of all these modalities of pre-erythrocytic malaria vaccines.

## Materials and methods

### DNA constructs

TRAP and CSP protein sequences are from *P*. *berghei* strain ANKA (accession XP_677379.2 for TRAP and XP_022712148.1 for CSP) and from *P*. *falciparum 3D7* (XP_001350088.1 for TRAP and XP_001351122.1 for CSP). Numbering is for the immature sequence. Synthetic cDNAs were codon optimized for mammalian expression by Atum Bio (Newark, CA) for *P*. *berghei* constructs and by GenScript (Piscataway, NJ) for *P*. *falciparum* constructs. *P*. *berghei* chimeras and TRAP-N constructs contain TRAP amino acids Gln^25^- Pro^291^, with amino acids 99–119 (KRYGSTSKASLRFIIAQLQNN) in the I domain α2 and α3 helices replaced by the equivalent sequence from *P*. *falciparum* (HSDASKNKEKALIIIKSLLST) [16] due to unsuccessful expression of the native *Pb*TRAP sequence. *P*. *berghei CSP* fusion fragments contain CSP amino acids Asn^164^- Ser^318^ and Pro^239^-Ser^318^ for TRAP-N/CSP-RT (GenBank deposition MN43370) and TRAP-N/CSP-T chimeras (GenBank deposition MN433699), respectively, with Asn^280^ mutated to Ser to remove the putative N-glycosylation site. *P*. *falciparum* chimeras contain TRAP amino acids Arg^26^-Asp^297^ with the nonconserved Cys^55^ and the N-glycosylation site Asn^132^ mutated to Ser and Gln, respectively, and CSP Gln^96^- Ser^375^ and Asn^207^- Ser^375^, for the TRAP-N/CSP-JRT (GenBank deposition MN433701) and TRAP-N/CSP-RT (GenBank deposition MN433702), respectively. *P*. *berghei* constructs were inserted in pLexM vector [16]. *P*. *falciparum* constructs were cloned into Nhe I-Bam HI sites of the pIRES2-EGFP vector (Takara Bio, formerly Clontech). All constructs contain a modified murine kappa chain secretion signal peptide and C-terminal His tag [16], and were confirmed by DNA sequencing.

### Hybridomas to TRAP

To elicit and characterize monoclonal antibodies (mAb), we used previously described [16] *P*. *falciparum* (strain NF54 clone 3D7) TRAP constructs containing amino acid residues 41–240, and 26–299 and a full length ectodomain construct, residues 26–511, cloned in the same vector. The non-conserved C55 and N-glycosylation sites were mutated in all three constructs (C55G, S132N, S477N, and N483S). For cell surface expression, the TRAP ectodomain construct utilized a murine kappa chain secretion signal peptide, an N-terminal Flag tag (DYKDDDDK) and a C-terminal glycosylphosphatidylinositol (GPI) attachment signal peptide from CD55/DAF (decay accelerating factor), GTTSGTTRLLSGHTCFTLTGLLGTLVTMGLLT. The construct was stably expressed in L1.2 cells (murine B lymphoma cell line). Cell surface expression was confirmed by flow cytometry using Flag M2 antibody (Sigma).

CB6F1 mice (Charles River Laboratories) were immunized intraperitoneally once with 20 μg purified TRAP 26–299 in Complete Freund’s Adjuvant (CFA) and twice in Incomplete Freund’s Adjuvant (IFA) two weeks apart (CL2 and CL5 fusions), or with L1.2 transfectants (10^7^ cells in 200 μl PBS), three times two weeks apart. Mice were boosted intraperitoneally 3 days before fusion with 20 μg protein or 10^7^ cells in PBS. In each fusion, spleen cells from 2 immunized mice were fused with myeloma P3X63Ag653 cells. Hybridoma supernatants were screened first by ELISA coated with either purified TRAP 26–299 or 26–511. Positives from ELISA were then screened by fluorescence flow cytometry (FACS) using L1.2 transfectants expressing the GPI-linked TRAP. Positive hybridoma supernatants were further tested for staining *P*. *falciparum* sporozoites dried on slides (Sanaria, Rockville, MD). Sporozoites were hydrated with PBS, permeabilized with 0.1% Triton in PBS, and incubated with hybridoma supernatants, followed by Alexa Fluor 647-labeled anti-mouse IgG.

For antibody purification, hybridoma cells were cultured in DMEM containing 6% ultra-low IgG FBS (Sigma). Antibodies were purified using protein G affinity chromatography and biotinylated using EZ-link sulfo-NHS-LC biotin reagent following manufacturer’s instruction (Thermo Scientific).

### Other antibodies

Antibodies 1G12, 4B3 and 4C2 to *P*. *falciparum* CSP were kindly provided by Dr. Nicholas J. MacDonald (NIH, Malaria Vaccine Development Branch). 3D11 and 2A10 hybridomas to *Pb* and *Pf* CSP repeat regions, respectively, were obtained from BEI Resources (ATCC), and IgG antibodies were purified from serum-free culture supernatants using protein G affinity chromatography. Secondary antibodies were rabbit polyclonal anti-His (Delta Biolabs), Horseradish peroxidase (HRP)-anti-rabbit and HRP-anti-mouse IgG (GE Healthcare) for Western blot, and HRP-penta-His antibody (Qiagen) and HRP-anti-mouse IgG (Abcam) for ELISA.

### Cell culture

293T cells (ATCC) were cultured in DMEM medium supplemented with10% fetal bovine serum (FBS). 293S GnTI- cells [27] and Expi293F cells (Thermo Fisher Scientific) were cultured in suspension [28] in serum-free Ex-Cell 293 medium (Sigma) and Expi293 medium (Thermo Fisher Scientific), respectively.

### Protein expression and purification

293T cells in 6-well tissue culture plates were transfected using Lipofectamine^2000^ according to manufacturer’s instruction (Thermo Fisher Scientific). For scaleup transient transfection of 293S GnTI^-^ cells, suspension cultures were transfected using polyethyleneimine [29]. Culture supernatants were harvested 6 days later. For stable transfection of Expi293F cells, adherent Expi293F cells were transfected in DMEM medium with 10% FBS using Lipofectamine^2000^. Selection was started 48 hours later by addition of 0.5 mg/ml G418 (final concentration). After 10–12 days, cells were harvested and sorted for top 5% GFP positive cells on a FACSAria (BD Biosciences). Cells were sorted a 2^nd^ time to further enrich GFP expressing cells. Sorted cells were expanded in suspension culture in serum-free Expi293 medium for protein purification. Proteins were purified from culture supernatants of transfectants by Ni-NTA followed by gel filtration chromatography as described [16]. For endoglycosidase H (Endo H) treatment, Ni-NTA purified materials were buffer exchanged to 50 mM sodium acetate, pH 5.5, and 150 mM NaCl, and digested with Endo H at 1:20 mass ratio of enzyme:protein at 4°C, overnight. Fractions from gel filtration chromatography were subjected to non-reducing SDS-PAGE and monomeric protein peak fractions were pooled and stored in aliquots at -80°C.

### Western blot

Culture supernatants (10 μl) from transiently transfected 293T cells were mixed with 2.5 μl 5x Laemmli sample buffer containing 25% β-mercaptoethanol or 25 mM N-ethylmaleimide for reducing and non-reducing SDS-polyacrylamide gel electrophoresis (PAGE), respectively. Blotting to PVDF membrane was carried out using Tran-Blot Turbo transfer system (Bio-RAD). Membrane was probed with 0.4 μg/ml primary antibody, followed by incubation with HRP-conjugated 2^nd^ antibodies and chemiluminescence imaging using LAS-4000 system (Fuji Film). ImageJ software was used for quantitation of protein bands.

### Multi-angle light scattering analysis (MALS)

Purified protein was subjected to gel filtration with a Superdex 200 10/300 GL column (GE Healthcare Life Sciences) in 30 mM Tris-HCl, pH 8, 350 mM NaCl using an Agilent liquid chromatography system, a DAWN HELEOS II multi-angle light scattering detector, an Optilab T-rEX refractive index detector, and UV detector (Wyatt Technology Corporation). Data were processed in ASTRA 6 using the protein conjugate model. The dn/dc value of 0.185 ml/g was used together with A280 extinction value calculated from protein sequence.

### mAb characterization with enzyme-linked immunosorbent assay (ELISA)

96-well ELISA plates (Costar) were coated with 50 μl of purified antibodies at 5 μg/ml in 50 mM sodium carbonate buffer, pH 9.5, 50 μl/well for 2 hrs at 37°C, and blocked with 3% BSA for 90 min at 37°C. His tagged TRAP or TRAP-CSP fusion proteins (50 μl, 0.4 μg/ml) was added and incubated at 4°C overnight. Binding was detected with HRP-anti-His (Penta-His Ab at 1:5000 dilution) or with biotin-labeled primary antibody at 0.5 μg/ml followed by HRP-streptavidin. 10 min after addition of peroxidase substrate (Life Technologies), plates were read at 405 nm on an Emax plate reader (Molecular Devices).

### Immunization

10 μg *P*. *berghei* TRAP/CSP fusion antigens diluted in 100 μl PBS were mixed with 100 μl AddaVax adjuvant (InvivoGen, San Diego), and completely emulsified by pushing the mixture between two glass syringes through an 18 ga connector with two female Luer lock adapters. BALB/c mice (The Jackson Laboratory) were injected intraperitoneally (i.p) with 200 μl of antigen and adjuvant emulsion per mouse. Mice were immunized 3 times at intervals of three weeks. Control mice received PBS and adjuvant emulsion. Tail blood yielding 50–100 μl serum was collected from each mouse for measuring antibody responses and stored at -80°C.

### Antibody titer measurement

96-well ELISA plates were coated with 50 μl of TRAP or TRAP-CSP fusion proteins at 2.5 μg/ml in sodium carbonate buffer, pH 9.5, overnight at 4°C. Plates were blocked with 3% BSA. Sera were serially diluted 5-fold starting from 1:200, and 50 μl/well added in duplicate and incubated for 2 hrs at room temperature. Positive control antibodies 3D11 to the *P*. *berghei CSP* repeat region and to the His tag at the C-terminus of the antigens were used at 1 μg/ml. Serum from adjuvant alone immunized mice was diluted and added to each plate as negative control. After incubation with HRP-anti-mouse whole IgG, peroxidase substrate was added, and absorbance at 405 nm was read 10 min later. A semi-logarithmic dilution curve (x-axis: log dilution and y-axis: OD_405_, subtracted by the negative control) was generated for each serum sample, and a line parallel to the x axis was drawn at half of the OD value of the 3D11 or His tag positive control antibodies. Antibody titer was taken as the dilution factor at which OD value was half of the positive control.

### Challenge with *P*. *berghei*-infecte*d* mosquitoes

PbGFP_CON_, a recombinant *Plasmodium berghei* (ANKA strain) that constitutively expresses GFP was used [30]. To infect mosquitoes, PbGFP_CON_-infected mice (3–7% parasitemia) were anaesthetized and laid over a cage of female *Anopheles stephensi* mosquitoes (50–100 mosquitoes per cage), which were allowed to feed for 15 min. Successful mosquito infection was confirmed 10 days later in dissected midguts by fluorescent microscopy detection of the presence of oocysts. At day 20 after blood feeding, infected mosquitoes (prevalence of infection >80%) were used to challenge immunized and naive mice described above. Mice were monitored for blood stage infection at days 7, 9 and 12 post challenge by Giemsa staining of thin blood smears and microscopic examination, and parasitemia was determined as % infected red blood cells. Mice that remained blood stage parasite-free after 12 days were considered sterilely protected.

### Ethics statement

Animal work was conducted in accordance with and was approved by the Harvard Medical School Institutional Animal Care and Use Committee (IACUC) under protocol #05010. Animals were cared for in compliance with the U.S. Department of Agriculture (USDA) Animal Welfare Act (AWA) and the Public Health Service (PHS) Policy on Humane Care and Use of Laboratory Animals.

## Results

### TRAP-CSP fusion antigens

In TRAP, the I and TSR domains are required for sporozoite motility and host cell invasion [9, 31]. Moreover, the I domain is the target of potent protective CD8+ T cell responses elicited by TRAP or whole sporozoite immunization in mice [32–34]. In CSP the repeat region contains immunodominant B-cell epitopes recognized by sporozoite neutralizing antibodies in mice and humans, whereas the αTSR domain contains T-cell epitopes associated with protection in mice and humans [34–37]. RI at the junction between the N-terminal domain and the repeat region has recently been identified to contain epitopes for potent protective antibodies in mice and humans [38–40].

Design of TRAP-CSP fusions was based on the above immunological data as well as structural data. Since regions containing protective B and T cell epitopes as well as structurally characterized, well-folded domains lie at the N-terminal portion of TRAP and at the C-terminal portion of CSP, the orientation of these domains was preserved in the fusion proteins by fusing the N-terminal portion of TRAP to the C-terminal portion of CSP (Fig 1A and 1B). Our recently determined crystal structures defined the boundaries of the I and TSR domains of TRAP and the αTSR domain of CSP; these domains in their entirety were included in all fusion constructs to ensure proper folding [13, 16]. These portions were either joined directly (TRAP-N/CSP-T) or through a portion of the CSP repeat region corresponding to that included in RTS (TRAP-N/CSP-RT) in *Pb* and *Pf* fusions (Fig 1A and 1B). Additionally, a *Pf* fusion was made in which the CSP junction region and all repeats were included (TRAP-N/CSP-JRT) (Fig 1A and 1B).

On sporozoites, the TSR domains of TRAP and CSP can be C-glycosylated by attachment of mannose to a specific Trp residue and O-glycosylated by attachment of a fucose to a Thr residue and linkage of a hexose to the fucose. These modifications are reproduced in mammalian expression [13, 16, 41–43]. However, whether N-glycosylation occurs in sporozoites remains unknown [41, 44]. NX(S/T) N-glycosylation motifs were present in the TRAP but not CSP fragments used here. To investigate the immunogenic role of the four putative N-glycosylation sites in the *Pb* fusion proteins, they were expressed in GnTI^-^ cells as high mannose glycoforms, and tested with or without N-glycan shaving with Endo H to remove all but one N-acetylglucosamine residue from each N-linked high mannose glycan chain (dNG preparations). A single N-glycosylation site in the *Pf* fusion constructs was removed by mutating Asn^132^ to Gln in TRAP.

### Expression and purification of TRAP-CSP fusion proteins

Fusion proteins were expressed in mammalian 293 cells, as previously used to obtain *P*. *falciparum* and *P*. *vivax* TRAP and CSP fragments for crystal structures and carbohydrate determination [13, 16]. Western blotting of 293 transfectant culture supernatants showed that among *Pb* protein constructs (Fig 1C), the TRAP-N/CSP-RT fusion was expressed the best, followed by the TRAP-N/CSP-T fusion, and that the TRAP-N and CSP-RT components were expressed less well. Among *Pf* protein constructs (Fig 1D), TRAP-N/CSP-JRT was expressed best and TRAP-N and TRAP-N/CSP-RT were expressed less well. Expression of CSP-JRT was undetectable (Fig 1D, lanes 4 and 10). Thus, fusion to TRAP-N in TRAP-N/CSP-JRT rescued expression of CSP-JRT (Fig 1D, lames 3 and 9). *Pf*CSP-RT was also poorly expressed and was detected as a ~30 kDa band by antibody to the CSP repeats in reducing SDS-PAGE but not in non-reducing SDS-PAGE (Fig 1D middle panel, lanes 6 and 12), suggesting that heterogenous disulfide linkages lead to lack of detection of a single or few bands (Fig 1D middle panel, lanes 6 and 10). TRAP-CSP fusions were particularly highly expressed relative to the CSP component alone. This effect was most pronounced in non-reducing SDS-PAGE. Indeed, comparisons between results in non-reducing SDS-PAGE, which requires correct disulfide bond formation to show a monomer band, and reducing SDS-PAGE, which will show monomeric and multimeric disulfide-linked material equally well as reduced monomers, suggested that folding of the N-terminal TRAP moiety of fusion stabilized correct formation within the CSP moiety of disulfides, all of which localize to the αTSR domain. Thus, in *Pb*TRAP-N/CSP-RT, bands in reducing and nonreducing SDS-PAGE were comparable in intensity (Fig 1C lanes 4 and 9) while in *Pb*CSP-RT the band in reducing SDS-PAGE was stronger (Fig 1C lanes 5 and 10). Similarly, in *Pf*TRAP-N/CSP-JRT, the bands in reducing and non-reducing SDS-PAGE were similar in intensity, while no bands were evident for CSP-JRT at all (Fig 1D). Moreover, for *Pf*TRAP-N/CSP-RT, bands were similar in reducing and non-reducing SDS-PAGE, while a band for CSP-RT was only evident in reducing SDS-PAGE as described above (Fig 1D middle panel, band at ~30kDa). These results suggest that TRAP-N, when fused N-terminal to CSP fragments, may act as a chaperonin to enhance folding of the CSP moiety.

**Fig 2:**
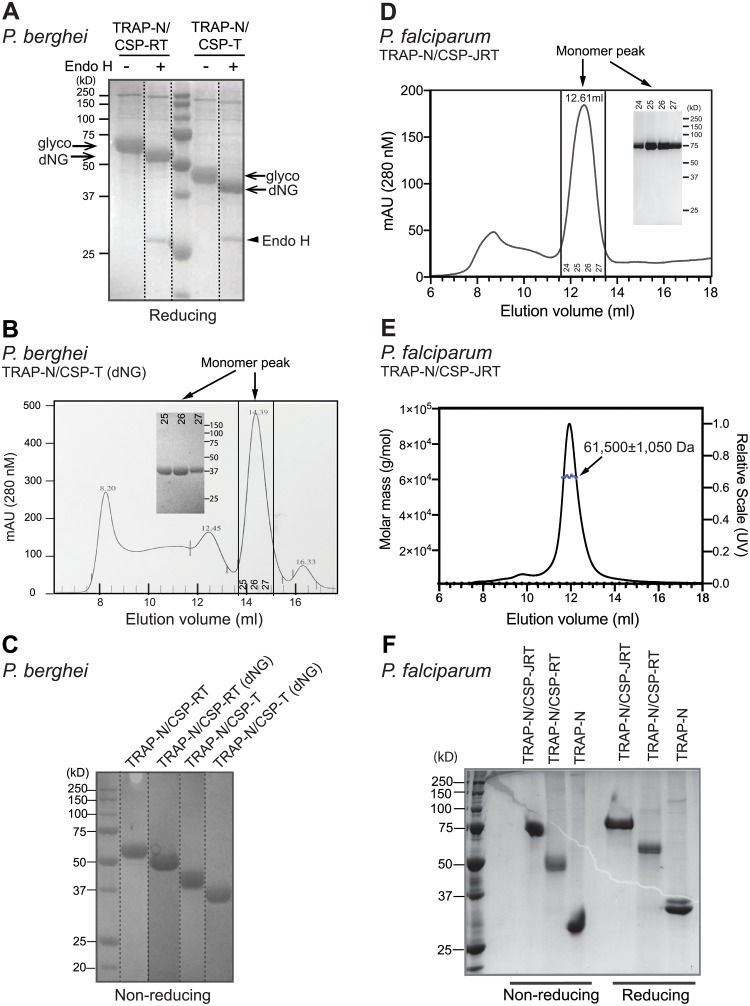
TRAP-CSP fusion protein purification. (A-C) *Pb* fusion proteins. (A) Fusions purified by Ni-NTA affinity chromatography were treated with or without Endo H and subjected to reducing SDS-PAGE and Coomassie blue staining. Arrows point to N-glycosylated (glyco) and Endo H-treated (dNG) bands and Endo H bands. Dotted lines separate lanes run on the same gel and moved. (B) Representative Superdex 200 10/300 GL purification of EndoH-treated, Ni-NTA purified TRAP-N/CSP-T. Inset: nonreducing SDS-PAGE of monomer peak fractions. (C) Nonreducing SDS-PAGE and Coomassie blue staining of *P*. *berghei* fusion proteins after Superdex S200 purification. Dotted lines divide lanes run on two identical gels and moved. (D-F) *Pf* fusion proteins. (D) Representative Superdex 200 purification of Pf TRAP-N/CSP-JRT. Inset: nonreducing SDS-PAGE of monomer peak fractions. (E) Multi-angle light scattering analysis of the peak material eluted at 12.61 ml in (D). (F) SDS-PAGE and Coomassie blue staining of Ni-NTA and S200 purified *Pf* fusion proteins and TRAP-N fragment run under nonreducing and reducing conditions.

Fusion glycoproteins were purified by Ni-NTA affinity chromatography (Fig 2A) followed by gel filtration (Fig 2B–2F). Purified *Pb*TRAP-N/CSP-RT and *Pb*TRAP-N/CSP-T proteins and their respective N-glycan shaved versions (designated dNG) each showed a single band by non-reducing SDS-PAGE (Fig 2C), confirming homogeneity and monomeric state. We also attempted to purify the two fusion components in isolation, *Pb*TRAP-N and *Pb*CSP-RT. However, purification yielded little if any material owing to low expression (Fig 1C) and possibly also due to misfolding of the CSP-RT component as described above. Well purified *Pf* fusion proteins and TRAP-N were also obtained (Fig 2F). Material from the putative monomeric peak in Fig 2D was further subjected to multi-angle light scattering (MALS) (Fig 2E). *Pf*TRAP-N/CSP-JRT has an experimentally measured mass of 61,580 Da, which is close to its theoretical protein mass of 60,800 Da, showing that it is monomeric. Adding the glycan mass of 780 Da for one residue each of mannose, fucose, and hexose on the TRAP TSR domain and fucose and hexose on the CSP αTSR domain [13, 16, 41–43] yields a theoretical glycoprotein mass of 61,580 Da. This theoretical glycoprotein mass is in excellent agreement with the experimental mass of 61,580±1,050 Da.

### mAb to *Pf*TRAP and native folding of TRAP-CSP fusions

**Fig 3:**
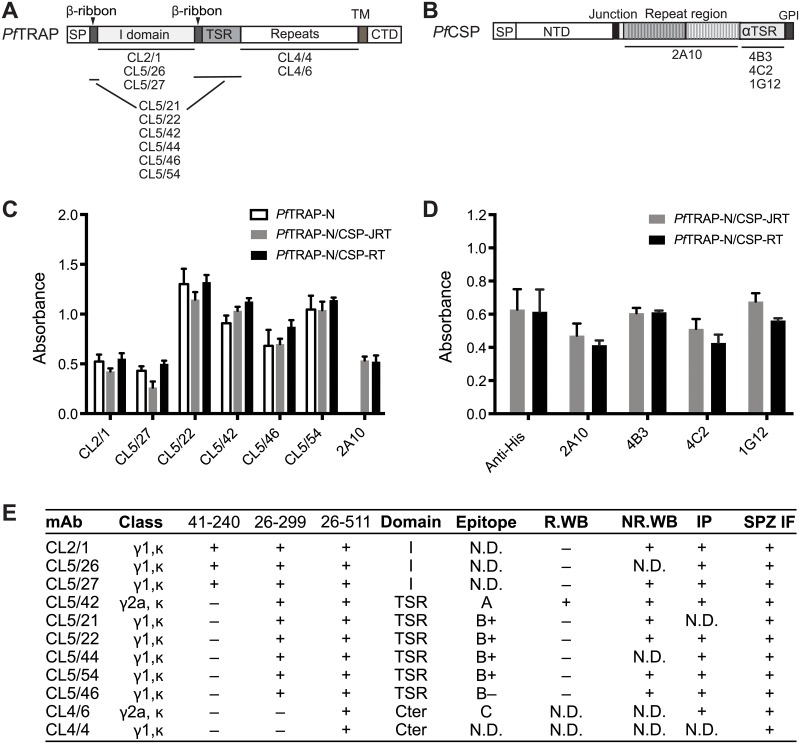
Reactivity of fusion proteins to antibodies that recognize correctly folded TRAP and CSP. (A-B) Schematic diagram of *Pf*TRAP (A) and *Pf*CSP (B) with monoclonal antibody epitopes. (C-D) Reactivity of conformation-dependent antibodies to domains within TRAP and CSP in the *Pf* fusion proteins. ELISA plates coated with indicated antibodies were incubated with antigens as described in Methods. Binding was detected with HRP-penta-His antibody (C) or biotin-labeled *Pf*TRAP conformation-independent antibody CL5/42 followed by HRP-streptavidin (D). Results are mean ± SD of triplicate wells, and are representative of 3 independent experiments. (E) Characteristics of antibodies to *Pf*TRAP. Mapping to domains in different fragments of TRAP is shown in (S1 Fig). Epitope mapping by competition among mAb is shown in S4 Fig. Reducing (R) and non-reducing (NR) Western blotting (WB) and immunoprecipitation (IP) are shown in S5 Fig. Representative sporozoite immunofluorescence (SPZ IF) is shown in S3 Fig.

To characterize the folding of *Pf* fusion proteins and to produce reference antibodies for future vaccine trials, we isolated and characterized 11 different mAbs to *Pf*TRAP (Fig 3). Binding of three mAb required the I domain, six mAb required additionally the TSR domain, and two mAb required the C-terminal portion of the TRAP ectodomain (Fig 3A and 3E and S1 Fig). All antibodies stained *Pf*TRAP transfectants and sporozoites (Fig 3E and S2 and S3 Figs). Competition among the antibodies for binding to LI.2 TRAP transfectants defined at least three epitopes. mAb CL5/42 uniquely recognized epitope A on the TSR domain and strongly bound in Western blotting both in absence and presence of disulfide reduction, showing that it recognizes its epitope both in folded and unfolded TRAP. Five antibodies recognized epitope B in the TSR domain; four of these antibodies completely cross-blocked one another (B+) while the antibody defining the B- epitope cross-blocked all the others but was only reciprocally cross-blocked by two of the others (Fig 3E and S4 Fig). mAb CL4/6 to the C-terminal region recognized distinct epitope C. Fluorescent staining of transfectants and sporozoites and immunoprecipitation showed that all eleven antibodies bind to native (folded) TRAP. All tested TRAP mAb except CL5/42 fail to work in Western blotting after reduction, showing that they are specific for the native, folded conformation.

*Pf*TRAP-CSP chimeras were characterized for native folding of their domains using our TRAP mAb and previously mapped CSP mAb [45] (Fig 3A and 3B). Antibodies to epitopes in all folded domains bound to *Pf*TRAP-N/CSP-JRT and *Pf*TRAP-N/CSP-RT fusion proteins at levels comparable to *Pf*TRAP-N (Fig 3C). Furthermore, conformation-specific mAb to the CSP αTSR domain reacted with the two fusion proteins at levels similar to a His tag antibody and the repeat region antibody 2A10 (Fig 3D). These results strongly suggest that the *Pf*TRAP I and TSR domains and the *Pf*CSP αTSR domain within the fusion proteins are correctly folded.

### Immune protection by TRAP-CSP fusion antigens

**Fig 4:**
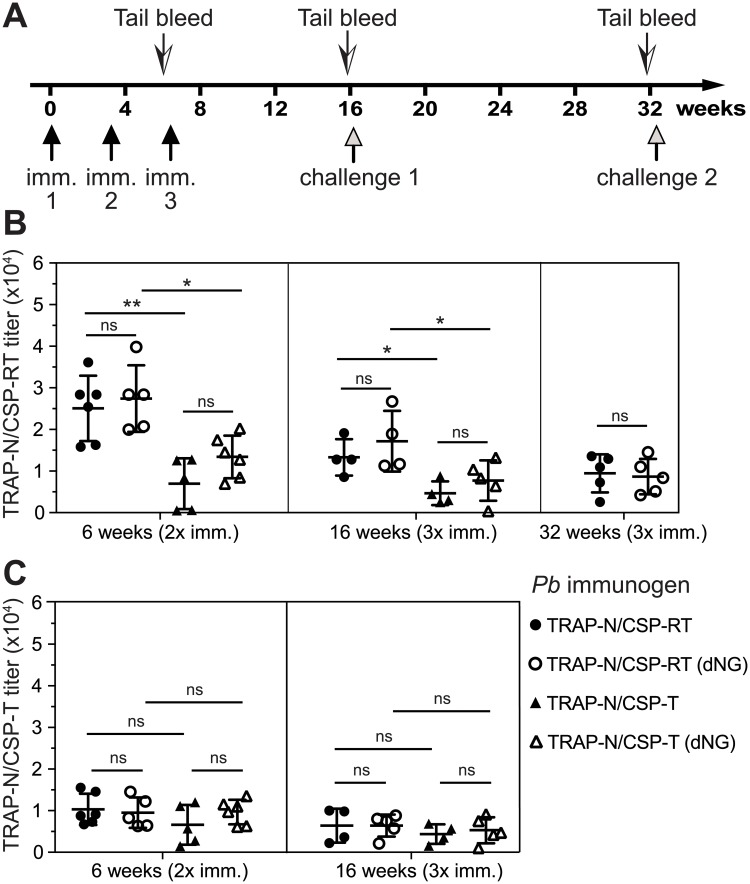
Vaccination timeline and antibody titers. (A) Timeline. (B and C) IgG titers against *P*. *bergei* TRAP-N/CSP-RT (B) and TRAP-N/CSP-T (C). Titers are shown for individual mice that survived to that time point and for which sera were obtained, together with mean and SD. *: P<0.05; **: P<0.01; ns: not significant by One-way ANOVA with Sidak’s multiple comparisons test (GraphPad Prism 7).

We tested *P*. *berghei* TRAP-N/CSP-T and TRAP-N/CSP-RT fusion antigens to evaluate immunogenicity and protection against infection. Mice were immunized three times with 10 μg of fusion antigens, with or without prior endoglycosidase H treatment to remove all but the Asn-linked N-acetylglucosamine residue of high-mannose N-glycans (dNG) in AddaVax, a squalene-based oil-in-water emulsion (Fig 4A).

IgG antibody titers were determined at 3 weeks after the second immunization, and to determine the durability of the humoral response, at both 10 and 26 weeks (6 months) after the third immunization. Mice were challenged with infected mosquitos at 16 weeks, and thus only those mice that were not infected (Table 1) were available for bleeding for antibody titer measurements and mosquito re-challenge at 32 weeks. TRAP-N/CSP-RT induced stronger immune responses than TRAP-N/CSP-T (Fig 4B). Titers, i.e. the inverse serum dilution giving half-maximal ELISA readings, averaged between 25,000 and 30,000 at 6 weeks after the second immunization for the fusion protein containing CSP repeats compared to between 7,500 to 15,000 for the fusion protein lacking the repeats. These differences in antibody titers were significant for both high mannose and dNG-glycoforms (Fig 4B, left). At 10 weeks after the third immunization, the titers were lower than after the second immunization (Fig 4B, middle panel), perhaps because of the greater elapsed time after the most recent immunization, i.e. 10 weeks after the third immunization compared to 3 weeks after the second immunization. Nonetheless, the differences between TRAP-N/CSP-RT and TRAP-N/CSP-T fusion proteins remained significant for both high mannose and dNG glycoforms. At 26 weeks after the third immunization, antibody titers were also measured, and were ~10,000 (Fig 3B, right panel). Titers were thus sustained in TRAP-N/CSP-RT-immunized mice for 6 months after the third immunization, although we cannot rule out boosting by challenge with *Pb* sporozoites delivered by mosquito bite 2.5 months after the third immunization (Fig 4A). No significant differences in IgG titers between high mannose and dNG glycoforms were seen for either fusion protein in any assay (Fig 4B and 4C).

**Table 1:** Protection against *P*. *berghei* challenge.

*P*. *berghei* fusion antigen	First challenge				Re-challenge of protected mice			
	Number infected mice	Parasitemia (%)			Number infected mice	Parasitemia (%)		
		Day 7	Day 9	Day 12		Day 7	Day 9	Day 12
TRAP-N/CSP-RT	0/5	0	0	0	0/5	0	0	0
TRAP-N/CSP-RT (dNG)	0/5	0	0	0	0/5	0	0	0
TRAP-N/CSP-T	3/5	3.5;2.9;2.3	3.8;4.7;3.9	8.4;10.4;8.5	2/2	2.4;3.1	3.8;4.6	9.1;10.5
TRAP-N/CSP-T (dNG)	3/5	2.6;2.4,1.6	3.2;2.8;3.1	10.9;12.5;13.9	2/2	2.7;2.9	3.2;3.7	13.9;12.6
None (naïve)	1/1	3.1	8.0	18.9	1/1	2.3	3.8	13.8

Mice were immunized in AddaVax adjuvant and challenged as shown in Fig 4A. RBC parasitemia is shown at the indicated number of days after challenge for each infected mouse; “0”: no infection of the entire group.

Greater humoral immune responses to TRAP-N/CSP-RT than TRAP-N/CSP-T might have been due to greater overall immunogenicity to the domains in common as well as to the CSP repeat region. Alternatively, the increased humoral response to TRAP-N/CSP-RT might have been directed to the only difference between the two fusion proteins, i.e. to the CSP repeats. To distinguish between these two possibilities, we compared antisera from all immunized mice for titers against both TRAP-N/CSP-RT (Fig 4B) and TRAP-N/CSP-T (Fig 4C). The results show that the antibody titers to the three folded domains in common between the immunogens were indistinguishable among the four groups of mice (Fig 4C). Therefore, all of the increase in the IgG titer of mice immunized with TRAP-N/CSP-RT compared to TRAP-N/CSP-T (Fig 4B and 4C) was due to antibodies to the CSP repeat region. Furthermore, quantitative comparison of the titers of mice immunized with TRAP-N/CSP-RT (titer ~26,000) and TRAP-N/CSP-T (titer ~ 10,000) showed that ~60% of the IgG immune response to TRAP-N/CSP-RT was directed to the CSP repeat region.

Balb/C mice were challenged 10 weeks after their third immunization by being bitten by *Pb*-infected *Anopheles stephensi* mosquitoes (Fig 4A). Monitoring blood stage infection showed that 5 out of 5 mice immunized with TRAP-N/CSP-RT and TRAP-N/CSP-RT (dNG) antigens were completely free of blood stage parasites (100% sterile protection) (Table 1). In contrast, after immunization with the TRAP-N/CSP-T and TRAP-N/CSP-T (dNG) antigens, 3 of 5 challenged mice in each immunized group showed blood stage infections (Table 1). As no differences were apparent between intact and endoglycosidase-treated (dNG) antigens in serum antibody titers or protection, we combined them as the TRAP-N/CSP-RT (comb) and TRAP-N/CSP-T (comb) groups. All TRAP-N/CSP-RT (comb) animals were protected (10/10) compared to only 4/10 TRAP-N/CSP-T (comb) animals (p = 0.011, Fisher’s exact test).

To evaluate maintenance of protection, sterilely protected mice were re-challenged 6 months after the last immunization (Fig 4A). Strikingly, the TRAP-N/CSP-RT and TRAP-N/CSP-RT (dNG) immunized groups were again completely protected (10/10) whereas no animals immunized with TRAP-N/CSP-T and TRAP-N/CSP-T (dNG) antigens were protected (0/4) (Table 1) (p = 0.001, Fisher’s exact test). We further analyzed the results by determining how many animals survived both the first and second challenge. The difference between the TRAP-N/CSP-RT (comb) (10/10) and TRAP-N/CSP-T (comb) (0/10) groups was highly significant (p<0.0001, Fisher’s exact test).

These results showed that immunization with TRAP-N/CSP-RT and its N-glycan shaved (dNG) version conferred sterile protection that was sustained for half a year after immunization, whereas TRAP-N/CSP-T and its dNG version, lacking the CSP repeat region, were less protective. Thus, the CSP repeat region enhanced protection, which correlated with higher antibody titers elicited by the TRAP-N/CSP-RT and TRAP-N/CSP-RT (dNG) fusion antigens (Fig 4B).

## Discussion

A fusion strategy combining TRAP and CSP fragments into a single fusion protein that contains well-folded protein domains and regions with protective T- and B-cell epitopes from each fusion partner has been shown here to yield a vaccine that stimulates sterilizing immunity. An important advance in our study is the demonstration that placing TRAP N-terminal to CSP in fusion proteins greatly improved production of well-folded, monomeric material. We expressed both *Pb* and *Pf*TRAP-CSP fusion proteins in a mammalian system that allows proper disulfide formation and O-fucosylation and C-mannosylation post-translational modifications. For protein-based subunit vaccines, proper folding and disulfide bond formation is critical to elicit antibody responses to native protein epitopes. Most targets of malaria vaccines, including TRAP and CSP, contain multiple disulfide bonds that are difficult to form properly using bacterial expression. Proper folding of the αTSR domain of CSP has been achieved in eukaryotes including yeast by using a secretion signal sequence to target it to the endoplasmic reticulum, where disulfide bond formation is catalyzed, after which CSP is secreted [13, 45].

In contrast, it appears unlikely that the CSP αTSR domain is properly folded in the RTS,S vaccine. RTS consists of half of the repeat region (R) and the αTSR domain (T) of CSP fused to the small hepatitis B virus surface antigen, sHBsAg, which is termed S [14]. To obtain a product that can be extracted from yeast and formulated, a 4-fold excess of free S, which is used as the hepatitis B vaccine, must be co-expressed in yeast to produce what is termed RTS,S. A precursor vaccine fused only the repeats of CSP to HBsAg, did not require co-expression with free S, and was well behaved as shown by electron micrographs of particles that look very similar to native HBsAg particles [46]. In contrast, no particle electron micrographs, SDS-PAGE, gel filtration, or other characterization data have been published for RTS,S, and the only published information on its extraction and purification from yeast is that “RTS,S was manufactured by SmithKline Beecham Biologicals according to approved standard operating procedures and manufacturing practices” [14]. RTS lacks a secretion signal sequence and thus is not targeted to the secretory pathway where disulfide bonds are formed. This fact, together with the good behavior of the HBsAg fusion with the repeats (which lack cysteines) and the poor behavior of RTS (which contains αTSR cysteines), as evidenced by the requirement for a 4-fold excess of S over RTS, suggests that RTS is not properly disulfide-bonded. We were unfortunately unable to test this hypothesis or compare RTS,S to our TRAP-CSP fusion proteins, because we were unable to obtain a sample from GlaxoSmithKline Biologicals, which manufactures and controls RTS,S, despite over $276 million to support its clinical development from the Gates Foundation. A slightly different fusion of RT to S termed R21 forms HBsAg-like particles and does not require S coexpression; however, R21 appears to form disulfide-linked multimers, because even after reduction, it shows dimeric as well as monomeric material in SDS-PAGE [24].

In contrast to RTS,S and R21, we have described here a glycoprotein vaccine that can be processed and secreted by any human or higher eukaryotic cell, and thus can not only be used directly as an immunogen, but also could be formulated as a nucleic acid or viral vector vaccine. We showed that mammalian expressed, purified monomeric *Pf*TRAP-CSP fusion proteins are properly folded by demonstrating their reactivity to a panel of antibodies that recognize correctly folded I and TSR domains of *Pf*TRAP and the αTSR domain of *Pf*CSP. Whether the αTSR domain of RTS,S and R21 is folded has not been demonstrated. We expect that the purified monomeric *P*. *berghei* TRAP-CSP fusion proteins, like those from *P*. *falciparum*, are properly folded.

Importantly, vertebrate cells mannosylate and fucosylate TSR domains similarly to *Plasmodium* sporozoites. X-ray crystallography and mass spectrometry have shown that the TSR domains of *Plasmodium* TRAP or its orthologue in *Toxoplasma gondii*, when expressed in mammalian cells, bear a C-linked mannose on tryptophan and O-linked fucose on threonine [16, 43]. Both modifications are also found on TRAP on the surface of *P*. *falciparum* sporozoites [41]. Furthermore, the αTSR domain of CSP expressed in mammalian cells is fucosylated [13], as is the αTSR domain of intact CSP on the surface of *P*. *falciparum* sporozoites [41]. Yeast lack an ortholog of PFUT2, the gene required for fucosylation of TSR domains, and the αTSR domain expressed in *Pichia* was not fucosylated [13]. Fucosylation and mannosylation of TSR domains in Apicomplexans have only recently been recognized [13, 16, 41, 43] and have not been previously appreciated in the malaria vaccine literature. However, these modifications may constitute important epitopes and enhance folding and expression, and hence immunogenicity, of vaccine antigens.

Less is known about N-glycosylation in *Plasmodium*. Compared to other eukaryotes, *Plasmodium* makes severely truncated N-glycan precursors composed of one or two N-acetylglucosamine (GlcNAc) residues and incorporates them in schizonts [44], yet these have not been detected in sporozoites [41]. We compared immune responses in mice to *Pb*TRAP-N/CSP-RT and *Pb*TRAP-N/CSP-T fusion proteins with high mannose N-glycans to fusion antigens with all but one N-acetylglucosamine residue removed by Endo H (dNG). We found comparable antibody responses and no difference in protection against *P*. *berghei* infection. N-glycosylation of the *Pf*s25 antigen delivered as DNA vaccine did not significantly affect antibody response and malaria transmission blocking efficacy [47]. In mixed results in an Aotus monkey trial of MSP1 antigen protection from *P*. *falciparum*, MSP1 purified from mouse milk with or without mutational removal of two N-glycosylation sites gave substantial protection or no protection, respectively, whereas N-glycosylated MSP1 purified after insect cell expression was protective [48]. Overall, the results from our and previous studies suggest that the impact of N-glycosylation of malarial antigens on protection efficacy may vary both among antigens and types of glycans.

The *Pb*TRAP-N/CSP-RT fusion antigen containing from TRAP the I and TSR domains and from CSP the C-terminal half of the repeat region and the αTSR domain conferred durable and complete sterile protection against *P*. *berghei* infection in BALB/c mice. By comparison, the *Pb*TRAP-N/CSP-T antigen that lacked the CSP repeat region was significantly less protective, showing that the repeat region provided enhanced protection, which correlated with higher antibody responses elicited by TRAP-N/CSP-RT than TRAP-N/CSP-T antigens. Identical results on sterilizing immunity and antibody responses were obtained with fusion antigens containing high mannose or Endo H-shaved N-glycans. Further studies are required to understand whether the I and TSR domains of TRAP and the repeats and αTSR domain of CSP separately or synergistically contribute to sterile protection by the *Pb*TRAP-N/CSP-RT fusion antigen.

Given the encouraging protection conferred by the *Pb*TRAP-N/CSP-RT fusion antigens, it is interesting that we demonstrated production in high yield of similar and longer *P*.*falciparum* fusion proteins. These fusions included *Pf*TRAP-N/CSP-JRT, which includes the junction region and all the repeats of *Pf*CSP; i.e., about twice as many as in *Pb*TRAP-N/CSP-RT and RTS,S. Recently identified potent and protective “dual specificity” antibodies to *Pf*CSP bind to the NANP repeats and the junction region [39, 40]. This region was not included in our *P*. *berghei* constructs or RTS,S but is present in *Pf*TRAP-N/CSP-JRT. Comparison of protective efficacy of *Pf*TRAP-N/CSP-JRT and PfTRAP-N/CSP-RT fusion proteins, which we obtained in highly pure and monomeric form, would provide insights into the role of the junction region in protection. In addition, we have succeeded in expression and purification of *Pf*TRAP and *Pf*CSP fragments (here and in [13, 16]), which would be important controls in further validating the fusion strategy.

Our strategy for design of TRAP-CSP fusion antigens from *P*. *berghei* and *P*. *falciparum* can apply to other *Plasmodium* species and be extended to design of further multi-antigen/multi-stage fusion antigens, for example in combined pre-erythrocytic and transmission-blocking vaccines. Mammalian expression has been developed into a much higher yield and cost effective process than it was in the 1980s and 1990s, when yeast expression of hepatitis and RTS,S vaccines was developed, and ensures proper disulfide formation and similar mannosylation and fucosylation of malarial antigens as in *Plasmodium*. Importantly, careful attention to the efficacy of vaccine antigen production in mammalian cells is also directly applicable to production of the same antigens when they are encoded by RNA, DNA, or viral vectored vaccines.

## Supporting information

S1 Text Supporting figure legends. (DOCX)

S1 Fig Epitope mapping. (EPS)

S2 Fig mAb binding to TRAP transfectants. (EPS)

S3 Fig mAb staining of sporozoites. (EPS)

S4 Fig Antibody competition. (EPS)

S5 Fig Effectiveness of antibodies in immunoprecipitation and in Western blotting after disulfide reduction. (EPS)
