# Supplementary material for: Design and assessment of TRAP-CSP fusion antigens as effective malaria vaccines
Source: PLoS One. 2020 Jan 22;15(1):e0216260. doi: 10.1371/journal.pone.0216260 (PMC6975556; doi:10.1371/journal.pone.0216260)
Supplement: S1 Text — (DOCX) [file pone.0216260.s001.docx]

**Supplemental figure legends**

**Figure s1**. **Epitope mapping**. (A) mAb binding to TRAP constructs with the indicated TRAP protein constructs. ELISA was with 50 µl of 1:5 diluted hybridoma supernatant or culture medium control. Mouse antisera against TRAP ectodomain (aa 26-511) at 1:2000 dilution was included as positive control. OD_405_ values are averages of triplicates after subtraction with the medium control and are representative of at least 3 independent experiments. (B) Antibody epitopes. TRAP domain organization is shown with sequence positions. mAbs are grouped to the domains/regions to which they bind. The assignments in (B) are shown here for comparison to the results on which they are based in (A) and are also shown in Fig. 3A.

**Figure s2. mAb binding to TRAP transfectants.** (A and B) L1.2 stable transfectants (A) and 293T transient transfectants (B) expressing GPI-anchored TRAP ectodomain (residues 26-511) with an N-terminal Flag tag were stained with hybridoma supernatants or culture medium as control at 1:2 and 1:20 dilution (A) or 1:5 dilution (B), followed by FITC-goat anti-mouse IgG. Antiserum at 1:2000 dilution (A) and anti-Flag at 2 µg/ml (B) were used as positive controls. Mean fluorescence intensity (MFI) is shown after subtraction of medium control MFI. Data are representative of at least 2 independent experiments.

**Figure s3. mAb staining of sporozoites**. Permeabilized *P. falciparum* sporozoites were stained with hybridoma supernatants diluted 1:2 in PBS, followed by Alexa Fluor 647 labeled anti-mouse IgG as described in Methods. CSP antibody 2A10 (10 µg/ml) was used as positive control. Fluorescence and bright field (B.F.) images were observed with a EVOS FL fluorescence microscope with a 20x objective lens. Images are representative of sporozoite staining in 2-3 independent experiments. Scale bar: 20 µm.

**Figure s4. Antibody competition**. (A-F) L1.2 stable transfectants expressing GPI-linked TRAP ectodomain (residues 26-511) were incubated with unlabeled antibodies at 40 µg/ml for 1 hour on ice. Cells were spun down, supernatant was removed, and without washing, were suspended in 10 µg/ml biotin-labeled antibody for 1 hour on ice. After 3 washes, cells were incubated with Alexa Fluor-488 conjugated to streptavidin, washed, and analyzed by flow cytometry. Mean fluorescence intensity (MFI) of each biotin-labeled antibody is shown atop of each graph with competing antibodies shown below. (G) Summary of antibody competition. +, competing; -, not competing.

**Figure s5. Effectiveness of antibodies in immunoprecipitation and in Western blotting after disulfide reduction**. (A) Immunoprecipitation. L1.2 stable transfectants expressing TRAP-GPI were lysed in 1%Triton-100, 20 mM HEPES, pH 7.4, 150 mM NaCl and protease inhibitors. Lysate from 5x10^6^ cells in 300 µl was mixed with 400 µl of hybridoma supernatant or buffer with 6 ug anti-Flag or control mouse IgG and incubated overnight at 4^o^C. Immune complexes were pulled down with protein G beads, eluted, and equal amounts of immunoprecipitates and lysate control (1/4 of input) was subjected to reducing SDS 10% PAGE and Western blotting using rabbit antisera to purified TRAP ectodomain (aa 26-511). Samples run on separate gels, probed and imaged at the same time, are separated by a dotted line. (B) Reactivity of TRAP antibodies in Western blotting after reducing (R) or non-reducing (NR) SDS-PAGE. 300 ng of purified TRAP protein (aa 26-299) was subjected to SDS-PAGE and Western blot with the indicated TRAP antibodies (1 µg/ml final concentration). Mouse IgG negative control, not shown, did detect any band. TRAP protein migrated more slowly after disulfide reduction.
